# Lower Extremity Biomechanics Are Altered Across Maturation in Sport-Specialized Female Adolescent Athletes

**DOI:** 10.3389/fped.2019.00268

**Published:** 2019-06-28

**Authors:** Christopher A. DiCesare, Alicia Montalvo, Kim D. Barber Foss, Staci M. Thomas, Kevin R. Ford, Timothy E. Hewett, Neeru A. Jayanthi, Andrea Stracciolini, David R. Bell, Gregory D. Myer

**Affiliations:** ^1^Division of Sports Medicine, The SPORT Center, Cincinnati Children's Hospital Medical Center, Cincinnati, OH, United States; ^2^Department of Athletic Training, Nicole Wertheim College of Nursing and Health Sciences, Florida International University, Miami, FL, United States; ^3^Department of Physical Therapy, High Point University, High Point, NC, United States; ^4^Biomechanics Laboratories and Sports Medicine Research Center, Mayo Clinic, Rochester, MN, United States; ^5^Department of Orthopedic Surgery, Mayo Clinic, Rochester, MN, United States; ^6^Department of Physiology and Biomedical Engineering, Mayo Clinic, Rochester, MN, United States; ^7^Emory Sports Medicine Center, Johns Creek, GA, United States; ^8^Emory University School of Medicine, Atlanta, GA, United States; ^9^The Micheli Center for Sports Injury Prevention, Waltham, MA, United States; ^10^Division of Sports Medicine, Department of Orthopaedics, Boston Children's Hospital, Boston, MA, United States; ^11^Department of Orthopaedic Surgery, Harvard Medical School, Boston, MA, United States; ^12^Division of Emergency Medicine, Department of Medicine, Boston Children's Hospital, Boston, MA, United States; ^13^Wisconsin Injury in Sport Laboratory, Department of Kinesiology, University of Wisconsin-Madison, Madison, WI, United States; ^14^Department of Orthopedics and Rehabilitation, University of Wisconsin-Madison, Madison, WI, United States; ^15^Department of Pediatrics, University of Cincinnati College of Medicine, Cincinnati, OH, United States; ^16^Department of Orthopaedic Surgery, University of Cincinnati College of Medicine, Cincinnati, OH, United States

**Keywords:** sport specialization, female, maturation, biomechanics, injury risk

## Abstract

Sport specialization is a growing trend in youth athletes and may contribute to increased injury risk. The neuromuscular deficits that often manifest during maturation in young, female athletes may be exacerbated in athletes who specialize in a single sport. The purpose of this study was to investigate if sport specialization is associated with increased lower extremity biomechanical deficits pre- to post-puberty in adolescent female athletes. Seventy-nine sport-specialized female adolescent (Mean ± SD age = 13.4 ± 1.8 years) basketball, soccer, and volleyball athletes were identified and matched with seventy-nine multi-sport (soccer, basketball, and volleyball) female athletes from a database of 1,116 female adolescent basketball, soccer, and volleyball athletes who were enrolled in one of two large prospective, longitudinal studies. The athletes were assessed over two visits (Mean ± SD time = 724.5 ± 388.7 days) in which they were classified as pre-pubertal and post-pubertal, respectively. Separate 2 × 2 analyses of covariance were used to compare sport-specialized and multi-sport groups and dominant/non-dominant limbs with respect to pubertal changes in peak knee sagittal, frontal, and transverse plane joint angular measures and moments of force recorded while performing a drop vertical jump task. The sport-specialized group were found to exhibit significantly larger post-pubertal increases in peak knee abduction angle (*p* = 0.005) and knee abduction moment (*p* = 0.006), as well as a smaller increase in peak knee extensor moment (*p* = 0.032) during landing when compared to the multi-sport group. These biomechanical changes are indicative of potentially compromised neuromuscular control that may increase injury risk pre- to post-puberty in sport-specialized female athletes. Consideration of maturation status may be an important factor in assessing the injury risk profiles of adolescent athletes who specialize in sport.

## Introduction

Sport specialization, or a year- or near year-round commitment to one sport at the exclusion of others (1), is becoming increasingly prevalent among pre-adolescent and adolescent athletes (2). This trend may be driven by a number of factors, including an overall decrease in unstructured physical activity (i.e., “free play”), an increase in structured activity among youth (3), and an increased pressure on youth athletes to excel in sport (4). The latter of these is underscored by the potential economic benefit of sport success [e.g., college scholarships, elite achievement, or high professional sports salaries (5, 6)] and the theoretical competitive advantage that deliberate practice might give youth athletes. These potential benefits are reinforced by the media and public perception (7) and the influence of coaches, parents, and peers (8). Consequently, there is concern that youth athletes are not only specializing in greater numbers, but also at earlier ages (7, 9), which can contribute to adverse outcomes in these athletes, such as psychological burnout and an increased risk of musculoskeletal injury (10, 11). Given the nearly 10-fold increase in female sports participation since the inception of Title IX (12), young female athletes may be specializing in sport at an increasing rate (2, 13, 14).

Recently, there has been an increased emphasis to discern the implications of early specialization in sport with the goal of educating practitioners and parents to ensure safe sport involvement in youth athletes (15, 16). Specialized athletes typically engage in a large volume of year-round, intensive, often technical or otherwise specialized, sport-specific training (10, 11), and as a result, sport specialization has been associated with an increased risk for overuse injury (2, 14, 17, 18). This increased risk may be related to the homogeneity of movements associated with highly specialized training regimens that repeatedly stress the same musculoskeletal tissues (19). For young, developing athletes, physiological immaturities in bone and connective tissue may not allow these individuals to adequately handle the homogenous and repetitive stresses that result from continual practice of a small set of sport-specific skills, which can lead to accelerated rates of fatigue and injury in this population (20, 21). This increased risk may also be due in part to compromised motor ability stemming from the inadequate development of or practice of motor skills that come alongside repetitive, non-variable practice of specialized sport movements. Multi-sport participation at a young age has been shown to improve gross motor competence and overall motor ability (22), and may lead to improved neuromuscular control (23) and more effective sport performance as exhibited through more optimal lower extremity biomechanics, as well as more balanced physiological responses to sport participation (24, 25). It therefore may be beneficial for young athletes to engage in a variety of sports and/or physical activities to facilitate more comprehensive physical and motor development.

Biomechanical risk factors that predispose sport-specialized youth athletes to overuse injury may be compounded by maturation. Developing female athletes, who are already at an increased risk of musculoskeletal injury relative to males (26, 27), may be especially susceptible to the factors underlying increased musculoskeletal injury risk relative to early sport specialization. Adolescent females are more likely than males to exhibit decreased neuromuscular control and aberrant biomechanics, particularly at the knee (28–30), that can lead to a decreased ability to modulate forces during dynamic movements that occur during sport, like landing (31) and cutting (32). Moreover, during maturation, these deficits persist and are often exacerbated by structural changes, such as increases in height, mass, height of the center of mass, etc., that can lead to increases in the magnitude of external forces experienced during dynamic activity (31, 33–35). These movement patterns can lead to increased risk for both acute and chronic knee injury in these athletes (29, 36).

Given the increased rate of sport specialization in youth athletes and its association with knee injury risk, and the potential for young, developing female athletes to be especially susceptible to increased injury relative to males, it is important to identify potential mechanisms that might amplify biomechanical risk factors in this group. Identification of these mechanisms may serve to educate parents, coaches, and other practitioners and improve prevention efforts targeting neuromuscular control during maturation. The purpose of this study was to examine the knee biomechanical changes that occur pre- to post-puberty in adolescent female athletes and the effect of sport specialization on these changes. The hypothesis tested was that sport specialization would be associated with increased propensity toward knee joint biomechanical changes that underlie an increased risk for musculoskeletal injury.

## Materials and Methods

### Participants

The cohort for this study was selected from a database of 1,116 female, adolescent basketball, soccer, and volleyball athletes (Mean ± SD age = 13.4 ± 1.8 years) who were enrolled in one of two large prospective, longitudinal studies that were conducted over the course of 4 years (29, 37). The athletes were 93.6% Caucasian, 3.0% African-American, 1.0% Asian, 0.2% Native American, and 0.1% Hawaiian, with 2.3% declining to or failing to report their ethnicity. Each testing session occurred at the beginning of the athletes' respective competitive sports season. Prior to data collection, the study protocol was approved by the Cincinnati Children's Hospital Medical Center Institutional Review Board (IRB 2008-0023 and IRB 2009-0602, respectively), and informed written consent, along with child assent, was obtained from participants and their parents or legal guardians if under 18 years of age.

### Data Collection

Testing consisted of participant characteristics, including questionnaires to determine anthropometric measurements, medical history, indicators of sport participation and pubertal development, as well as a three-dimensional biomechanical analysis of a drop vertical jump (DVJ) task. The same research assistant administered the questionnaires and obtained anthropometric measurements, and a trained research biomechanist collected the data from the biomechanical analysis using pre-specified standard operating procedures.

**Figure 1:**
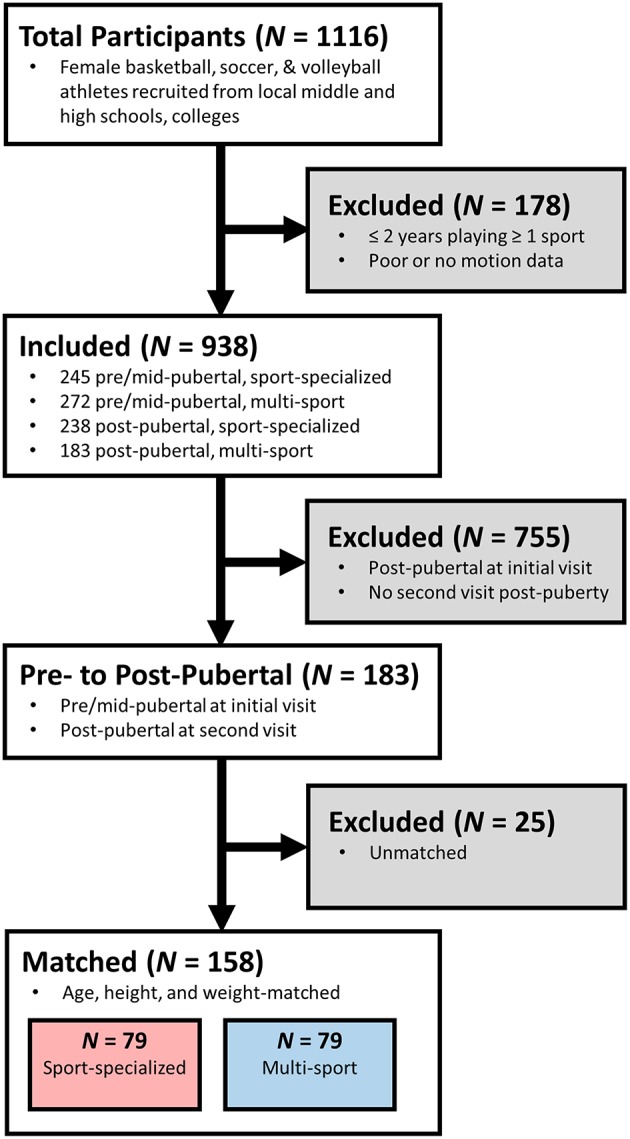
Flowchart illustrating the selection process for the cohort of sport-specialized and multi-sport athletes. Athletes were selected if they were classified as either “sport-specialized” or “multi-sport,” had longitudinal data (≥2 visits to the laboratory), were classified as “pre-pubertal” or “mid-pubertal” at their first visit, and had at least one longitudinal visit in which they were classified as “post-pubertal”.

From the initial cohort of 1,116, athletes were excluded if they were not classified as either sport-specialized or multi-sport as indicated from the sport participation questionnaire, had poor or missing biomechanical data, and who were classified as post-pubertal at the initial visit or did not have a follow-up visit in which they were classified as post-pubertal. After exclusion, 183 athletes met the criteria for inclusion into the study; athletes were selected such that there were equal numbers of sport-specialized and multi-sport athletes and then age-, height- and weight- matched based on their measurements at their initial visit, leaving 158 athletes in the final cohort (Figure 1). Each participant's initial and follow-up visits were separated by at least 6 months (sport-specialized M ± SD days = 773 ± 405, range = 293–1,827; multi-sport M ± SD days = 696 ± 373, range = 280–1,812).

#### Anthropometric Measurement

Height and weight were recorded for each participant using a standard medical scale, and body mass index (BMI) was computed from these measures. Each participant's dominant limb was also recorded by asking the participant which leg she would use to kick a ball as far as possible.

#### Sports Participation and Pubertal Questionnaires

During each testing session, both the sport specialization status and the maturational status of the athletes were assessed. Sport specialization status was determined by having participants complete a sports participation questionnaire, which asked athletes to report their participation in their current sport (i.e., basketball, soccer, or volleyball), as well as any additional sports, and the number of years in which they have participated in each sport. Participation was defined as having at least been a member of a competitive, organized team for an entire season in a given year. In the present study, participants were classified as “sport-specialized” if they had ≥2 years of participation in 1 sport and < 2 years participation in any other sports, and “multi-sport” if they had ≥2 years of participation in each of at least 2 sports. While the questionnaire did not include an assessment of year-round participation (i.e., ≥8 months out of the year) by single-sport athletes, which is a necessary component for classifying an athlete as truly sport specialized (1), this classification scheme was similar to that which was reported by Hall et al. (18), albeit differing slightly. Specifically, in contrast to Hall, single-sport athletes—regardless of the number of years they participated—were classified as “sport-specialized,” and athletes who competed in more than one sport were classified as “multi-sport.” The classification in the present study was used because of the ambiguity surrounding the sports participation questionnaire that was administered to participants. With respect to current sport involvement, the questionnaire did not differentiate between first-time athletes and athletes who had at least 1 year of participation (i.e., in both scenarios, their years of participation were recorded as “1”). Consequently, it was unclear at what point athletes who listed their number of years of participation as “1” began participating in that sport. As a result, the present study used 2 years of participation as the threshold for involvement in a given sport.

The pubertal status of each participant was determined with the modified Pubertal Maturation Observation Scale (PMOS) questionnaire (34, 38). The PMOS was developed by Davies et al. (38) as a clinician-friendly, unobtrusive tool to differentiate between pubertal stages without physical examination. This scale is based on several indicators of pubertal maturation, including growth spurt, menarcheal status, body hair, sweating, and muscular definition (39), and it can be used to reliably classify subjects into developmental stages based on a parental report and investigator observational report (38). The PMOS was completed by each participant's parent(s) or legal guardian(s). Positive answers to each of the questions in the PMOS were scored as a point, with ≤1 points, 2–4 points, and ≥5 points used to classify participants as “pre-pubertal,” “mid-pubertal,” or “post-pubertal,” respectively (40). To investigate the biomechanical changes to female athletes through maturation, athletes were selected if their maturational status was classified as being either “pre-pubertal” or “pubertal” during their first visit to the laboratory and they had at least one follow-up visit during which they were classified as “post-pubertal” based upon PMOS score (i.e., their maturational status changed between the time of their first visit and some future visit) (34). If the participants had more than one longitudinal visit in which they were classified as “post-pubertal,” the first visit in which they reached “post-pubertal” status was selected for analysis. Using these classifications, at the time of their first testing session, athletes were required to be classified as “pre-pubertal” or “pubertal,” and at the time of the second testing session, they were required to be classified as “post-pubertal.” Sport specialization status did not change between testing sessions for any participants.

#### Biomechanical Analysis of the DVJ

Data were collected on participants with a standard biomechanical assessment that utilized three-dimensional motion analysis (41). Participants were first instrumented with 37 retroreflective markers with a minimum of three tracking markers per segment. Markers were placed on the lower back between the S5 and T1 vertebrae, and bilaterally on the acromio-clavicular joint, lateral epicondyle of the elbow, mid-wrist, anterior superior iliac spine, greater trochanter, mid-thigh, medial and lateral femoral condyles, tibial tubercle, lateral and distal aspects of the shank, medial and lateral malleoli, the heel, the dorsal surface of the midfoot, the lateral foot (fifth metatarsal) and central forefoot (between the second and third metatarsals). A 10-camera, high-speed, passive optical motion capture system (Motion Analysis Corp., Santa Rosa, CA) sampled at 240 Hz was used to record the three-dimensional marker trajectories from each participant. Ground reaction forces in Newtons were collected with two embedded force platforms (AMTI, Watertown, MA) sampled at 1200 Hz that were synchronized with the motion capture system.

Before dynamic motion trials were collected, a static trial was conducted in which the participant was instructed to stand in anatomical pose with foot direction and placement standardized to the laboratory's global coordinate system to define the participant's neutral kinematic posture. Participants then performed a minimum of three trials of a drop vertical jump (DVJ) task, a commonly used motor task in lower extremity biomechanical assessments (29, 36, 42). During the DVJ, participants positioned themselves on top of a 31-cm box with their feet aligned with tape placed at the edge of the box, situated approximately shoulder-width apart. Participants were instructed to drop off the box with both feet at the same time, land on the force platforms in front of the box, and immediately perform a maximum effort vertical leap to attempt to grasp a maximally positioned overhead target. Each trial was performed with minimal rest in between (10–15 s). Trials were repeated if the participant did not leave the box with both feet at the same time or paused upon landing before performance of the maximum vertical leap, and the participant kept performing trials until three acceptable trials were obtained.

#### Data Processing

Marker trajectories and ground reaction forces were filtered using a low-pass, fourth-order Butterworth filter with a cutoff frequency of 12 Hz. A six-degree-of-freedom skeletal model was applied to the marker data to determine the position and orientation of all segments at each time sample, and the model was scaled to the participant's height and weight. Three-dimensional lower extremity joint angles were calculated with an XYZ Cardan rotation sequence, and ground reaction forces were used to calculate joint moments of force using an inverse dynamics analysis in Visual3D (C-Motion, Inc., Germantown, MD) and were referenced to coordinate axes systems about the proximal limb. Joint angles and moments were extracted from the stance phase, which was defined as the period of time from when subjects made initial contact with the force platforms (determined when the normal ground reaction force exceeded 10 N) until toe-off occurred. The joint angles and moments were then time-normalized to 101 data points (representing 0–100% of stance) using custom MATLAB (MathWorks, Inc., Natick, MA) software. Each participant's time-normalized waveforms were averaged across the three DVJ trials for each joint and plane of motion. In addition, the stance phase was further divided into the landing and propulsion sub-phases, respectively, differentiated by the point during normalized stance at which the participant's center of mass reached a minimum vertical height. Peak knee kinematic (joint angular motion) and kinetic (joint moments of force) measures in both the dominant and non-dominant limbs were extracted during the time-normalized, meaned landing sub-phase.

### Statistical Analysis

One-sample Kolgomorov-Smirnov (K-S) tests for normality were conducted for anthropometry and knee biomechanical measures at each visit. Independent *t*-tests were used to ensure no differences existed between the sport-specialized and multi-sport groups in age, height, weight, BMI, and sport participation years at either the initial testing session (”pre-pubertal”) or follow-up testing session (“post-pubertal”). Average years of sport participation was determined by summing the total number of years reported participating in all sports divided by the number of unique sports.

Post-pubertal knee kinematic and kinetic differences between sport-specialized and multi-sport athletes were determined using separate 2 × 2 (group × limb) analyses of covariance (ANCOVA) with their pre-pubertal measures and days between testing sessions being included as covariates to the model. Days between testing sessions was included as a covariate because of the wide time range (i.e., 280–1,827 days) between visits; this occurred because while many participants had multiple visits to the laboratory, only the first visit in which the participant was classified as “post-pubertal” was used in the analysis, which did not occur at the same time for all participants. Bonferroni corrections were used to control for multiple comparisons, and an alpha level of.05 was selected *a priori* to indicate statistical significance.

## Results

Table 1 describes the mean age, height, weight, BMI, and years of sport participation recorded for the sport-specialized and multi-sport groups during both testing sessions. Age, height, weight, and average sport participation were all found to be normally distributed (K-S; all *p* > 0.05), and no differences existed between any of these measures at the time of either testing session (*t*-test; all *p* > 0.05).

**Table 1:** Mean ± SD pre- and post-pubertal anthropometry and sport participation years in the sport-specialized and multi-sport groups.

	**Pre-pubertal**		**Post-pubertal**	
	**Sport-specialized**	**Multi-sport**	**Sport-specialized**	**Multi-sport**
Age (years)	12.2 ± 0.8	12.3 ± 0.9	14.3 ± 1.2	14.2 ± 1.1
Height (cm)	155.0 ± 7.7	155.0 ± 7.5	1.6 ± 5.2	1.6 ± 5.9
Weight (kg)	47.1 ± 10.1	47.6 ± 10.7	55.9 ± 7.5	55.7 ± 8.7
BMI (kg × m^−2^)	19.4 ± 3.0	19.5 ± 3.0	21.2 ± 2.8	21.1 ± 2.4
Sport participation (years)	4.5 ± 1.4	4.3 ± 2.1	6.2 ± 2.1	6.0 ± 2.3

**Figure 2:**
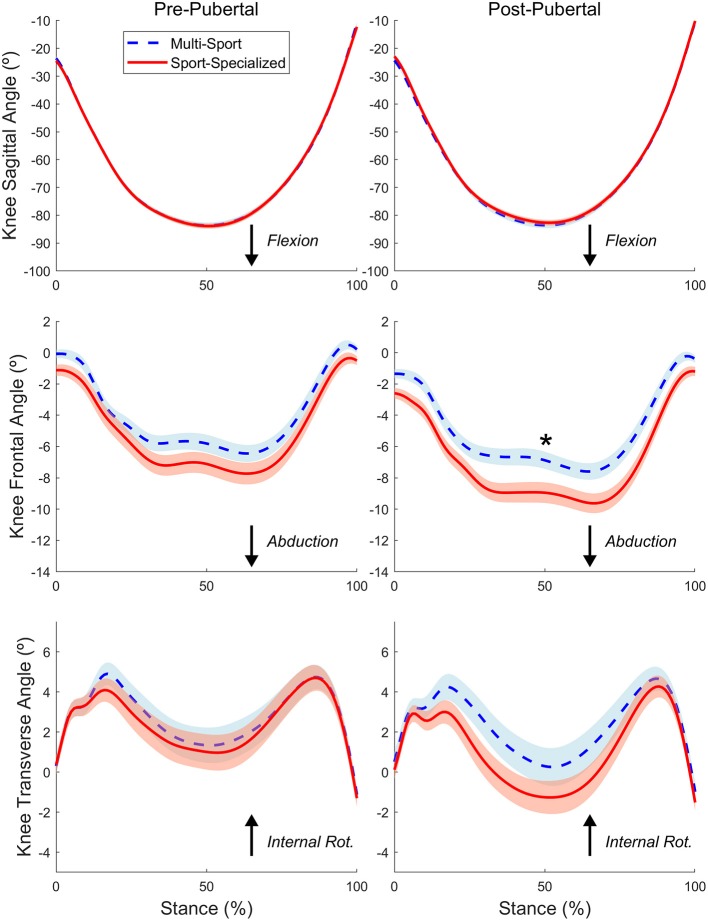
The time-normalized knee joint angular motions in the sagittal (Top), frontal (Middle), and transverse (Bottom) planes during the stance phase of the DVJ task for the sport-specialized (red) and multi-sport (blue) groups pre (Left) and post-puberty (Right). The waveforms represent the average joint angular motions between dominant and nondominant limbs. The shaded region represents standard error of the mean. “*” indicates statistically significant differences pre- to post-puberty.

**Figure 3:**
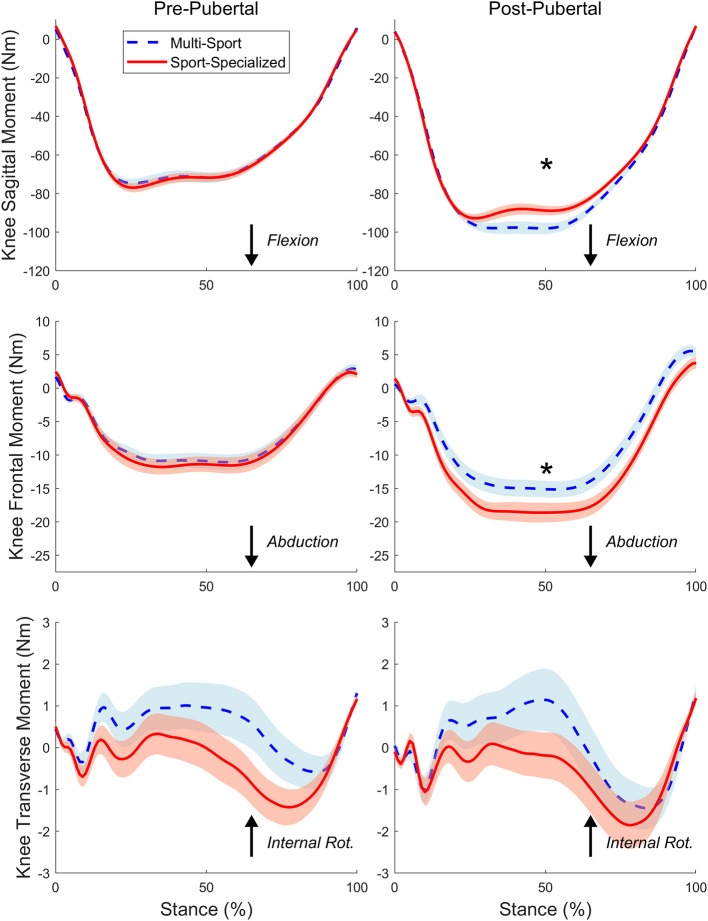
The time-normalized knee joint moments of force in the sagittal (Top), frontal (Middle), and transverse (Bottom) planes during the stance phase of the DVJ task for the sport-specialized (red) and multi-sport (blue) groups pre (Left) and post-puberty (Right). The waveforms represent the average net external moments between dominant and nondominant limbs. The shaded region represents standard error of the mean. “*” indicates statistically significant differences pre- to post-puberty.

**Figure 4:**
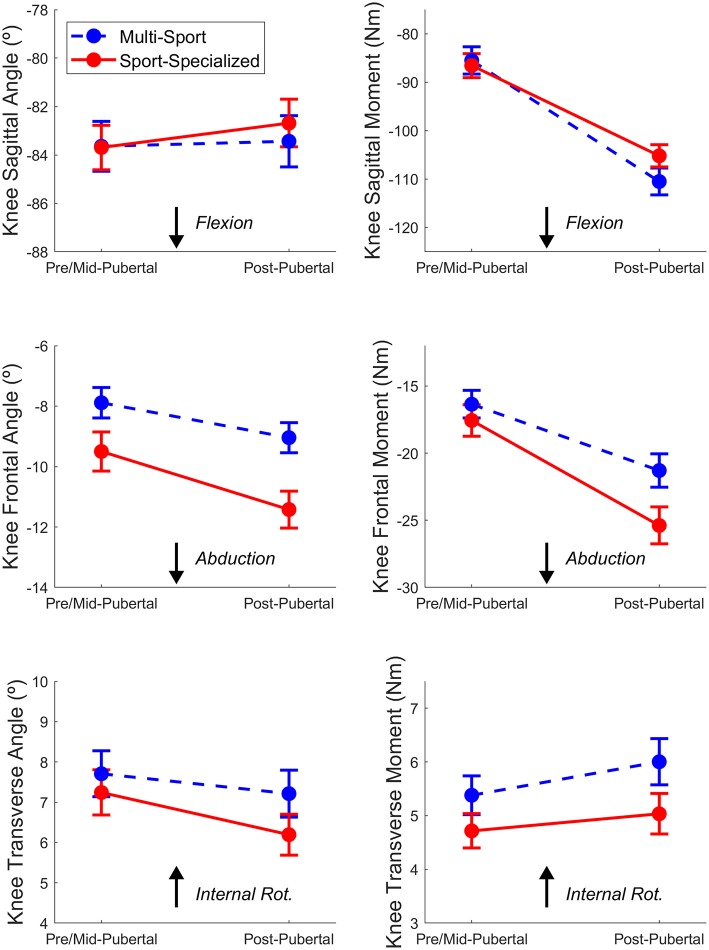
Mean peak knee kinematic (Left) and kinetic (Right) measures in the sagittal (Top) frontal (Middle), and transverse (Bottom) planes during the stance phase of the DVJ task for the sport-specialized (red) and multi-sport (blue) groups. The error bars represent standard error of the mean.

Figures 2, 3 show the mean time-normalized waveforms for the dominant limb knee joint moments of force, respectively, for the sport-specialized and multi-sport groups pre- and post-puberty. The mean peak knee kinematic and kinetic measures for both groups and limbs pre- and post-puberty are shown in Table 2. All knee kinematic and kinetic measures were found to be normally distributed (K-S; all *p* > 0.05) except for transverse plane knee joint moment (*p* = 0.011), which had moderate positive skewness; this variable was subsequently square-root-transformed before being submitted to ANCOVA. The analysis revealed significantly larger post-pubertal increases for the sport-specialized group in peak knee abduction angle [*F*_(1, 310)_ = 8.077, *p* = 0.005] and knee abduction moment [*F*_(1, 310)_ = 7.807, *p* = 0.006]. In addition, the sport-specialized group exhibited a significantly smaller increase in knee extensor moment [F(1, 310) = 4.616, *p* = 0.032] (Figure 4). No other main effects were observed the other kinematic and kinetic measures for either group or limb, or the group × limb interaction (all *p* > 0.05).

**Table 2:** Mean ± SD pre- and post-pubertal peak knee kinematic and kinetic measures in the sport-specialized and multi-sport groups.

	**Pre-pubertal**		**Post-pubertal**	
	**Sport-specialized**	**Multi-sport**	**Sport-specialized**	**Multi-sport**
Dominant limb				
Knee flexion (°)	−83.2 ± 8.2	−82.9 ± 9.2	−82.1 ± 9.0	−82.7 ± 9.6
Knee abduction (°)^*^	−8.9 ± 6.6	−7.6 ± 4.8	−11.3 ± 6.7	−8.6 ± 5.3
Knee internal rotation (°)	6.8 ± 5.7	7.0 ± 5.8	5.6 ± 4.4	6.5 ± 6.1
Knee extensor moment (Nm)^*^	89.3 ± 23.6	87.6 ± 27.1	107.3 ± 23.4	112.3 ± 28.0
Knee abduction moment (Nm)^*^	−15.2 ± 11.6	−13.9 ± 9.1	−23.8 ± 14.7	−19.6 ± 11.4
Knee internal rotation moment (Nm)	3.0 ± 2.8	3.6 ± 3.5	3.7 ± 4.5	4.4 ± 4.1
Non-dominant limb				
Knee flexion (°)	−84.2 ± 8.5	−84.4 ± 9.6	−83.3 ± 9.0	−84.2 ± 9.7
Knee abduction (°)^*^	−10.1 ± 6.2	−8.2 ± 5.5	−11.6 ± 5.1	−9.5 ± 5.1
Knee internal rotation (°)	7.6 ± 5.1	8.4 ± 5.7	6.8 ± 5.7	7.9 ± 5.3
Knee extensor moment (Nm)^*^	83.8 ± 22.5	83.4 ± 25.0	103.1 ± 21.8	108.7 ± 24.5
Knee abduction moment (Nm)^*^	−19.9 ± 11.1	−18.8 ± 12.8	−26.9 ± 12.6	−23.0 ± 14.2
Knee internal rotation moment (Nm)	6.4 ± 4.3	7.2 ± 4.3	6.4 ± 4.8	7.6 ± 5.1

* *Indicates statistically significant differences pre- to post-puberty*.

## Discussion

The aim of the present study was to examine the influence of sport specialization on knee injury risk biomechanics across puberty. The main finding of our study showed that sport-specialized female athletes exhibited knee kinematic and kinetic changes pre- to post-puberty that may increase risk for injury when compared to multi-sport female athletes (29). Specifically, the sport-specialized female athletes exhibited larger post-pubertal increases in peak knee abduction angle and knee abduction moment and a smaller increase in knee extensor moment during landing while performing the DVJ task than multi-sport athletes. The results indicate that female athletes who specialize in sport may amplify an increased risk for injury across puberty due to compromised neuromuscular control when compared to female athletes who chose not to specialize in early sport.

The competitive demands of many sports necessitate movement patterns and coordination strategies that accommodate high external forces experienced by adolescent athletes during dynamic activity. These external forces are magnified through maturation because of structural and inertial changes to the body, such as increases in mass, height, and segment length(s). In the present study, sport-specialized athletes exhibited larger post-pubertal increases in knee abduction angle and moment during landing when compared to multi-sport athletes. The relationship between sport specialization and knee abduction moment is particularly novel, as most previously described risk associations for sports specialization are for overuse injuries, such as patellofemoral pain (17, 18). However, demonstrating increased knee frontal plane moments associated with sport-specialized female athletes may have great implications in injury prevention of acute injuries such as anterior cruciate ligament tear, medial collateral ligament tear, and patellar instability. High knee abduction moments have been established previously as risk factors for both acute and chronic knee injury (29, 36) and thus, larger increases in this variable—as exhibited by the sport-specialized group across puberty—might be linked to increased injury risk. It is unclear what level of change in or magnitude of this variable can be considered meaningful as it pertains to knee injury; however, some studies differentiated athletes into low- and high-risk categories based on specified thresholds. For example, the maximum sensitivity and specificity to classify adolescent female athletes at high-risk for an ACL injury as having exhibited knee abduction moments greater than 25.25 Nm (29); in the present study, sport-specialized athletes exhibited average post-pubertal knee moments of 24 Nm and 27 Nm for the dominant and non-dominant limbs, respectively (as opposed to 19 Nm and 23 Nm, respectively, for the multi-sport group), which would classify these individuals at or near “high-risk” for ACL tear.

Prior studies investigating similar high-risk biomechanics in female athletes through maturation have shown maturation can underlie increased proliferation of lower extremity frontal plane mechanics that underlie increased injury risk. While these prior investigations did specifically examine sport specialization, the findings of this study support that the biomechanical changes through puberty that occur may in fact be compounded by early sport specialization (28–31, 33–35). This finding indicates that early sport specialization may be an additive factor for increased injury risk or that multi-sport diversity mitigates the development of insufficient neuromuscular control throughout the maturation process (28, 31). In addition, there is consistent evidence that indicates that youth should be involved in periodized strength and conditioning (e.g., integrative neuromuscular training) to help them prepare for the demands of competitive sport participation (24, 25). The current results may suggest that young females who specialize in a single sport can benefit from focused integrative neuromuscular training to enhance diverse motor skill development to reduce the proliferation injury risk factors particularly during maturational development (15, 23).

Sport-specialized athletes also experienced a smaller increase in knee extensor moment during landing. While over-reliance on the knee extensors during landing can be an indicator of decreased hip control and subsequent lower extremity injury risk (43), it can also indicate improved task performance, particularly during the DVJ (44). For the multi-sport group, these results (i.e., greater increase in knee extensor moment, smaller increase in knee abduction moment, and trend toward a greater increase in hip extensor moment (Figure 2) indicate that multi-sport athletes tend to exhibit more improved landing performance during the DVJ. These results may also indicate that multi-sport involvement may help adolescent athletes modulate and possibly ameliorate the inability to manage inertial demands that occur across puberty leading to decreased injury risk.

For young athletes, sport performance is greatly influenced by the amount of sport-specific practice and competitive sport participation. As a result, the perception among many coaches, parents, and athletes is that constant, and intensive training, beginning at a young age, will ultimately lead to significant sport achievement. However, currently there is no evidence to support the notion that early specialization in sport results in greater success. To the contrary, achieving elite status in sport has been shown to be related to young athletes participating in multiple organized sports, followed by sport specialization later in adolescence (5, 6, 45). Some individualized sports, like tennis, swimming, and gymnastics, involve early and intensive training by youth, and early sport specialization is becoming increasingly prevalent in some team sports such as basketball, soccer, and volleyball (13). Early childhood and adolescence are critical times during which diversification of movement is necessary for comprehensive motor and coordination development. In this light, early sport specialization may stifle the development of critical motor skills during childhood (46). Sport diversification during childhood and adolescence may promote improved motor competence (22), leading to greater sport specific skill and technique acquisition later in adolescence. The lack of established criteria for “early” sport specialization, and lack of consensus on training volume and age criteria add greatly to the challenges when trying to establish future guidelines (47, 48). Biomechanical analysis of movement patterns that might increase injury risk may help to guide sports participation for young specialized athletes.

The results of this study should be interpreted in light of the following limitations. The definition of sport specialization used to differentiate single- and multi-sport athletes. Our definition differed slightly from previous work (17, 18) and may not have accurately represented true sport specialization (i.e., year-round, single-sport participation to the exclusion of others); thus, the year-round component was not clear in the present study. However, given our modified definition, biomechanical differences were still able to be detected through maturation in sport-specialized female athletes. Future studies examining injury risk biomechanics across puberty in sport-specialized athletes should establish this more fully. In addition, the present study used pubertal characteristics to establish maturational stages. This classification scheme has been used previously (34); however the authors acknowledge that the usage of discrete pubertal classifications may have participants at the beginning or end of stages that creates a mix of maturational levels in the chosen groupings. The authors acknowledge that other metrics of maturation may be more biomechanically relevant to refine this classification (e.g., timing of peak growth height velocity, etc.) and thus, future work should explore these potential relationships. Future work should also examine both male and female sport-specialized athletes; the present study examined female athletes exclusively. As females tend to be more at risk for knee injury than their male counterparts, it may be that early sport specialization does not have as a profound of an influence on risky knee biomechanics in males.

In the present study, sport specialized female athletes exhibited altered biomechanics during landing while performing the DVJ task compared to multi-sport athletes. The results of this study suggest that the biomechanical changes that occur during maturation in specialized female athletes may be combinatory in injury risk profile development. The current definition of sport specialization in youth emphasizes early and continual (i.e., year-round) involvement in sport (47, 48). However, no consensus exists on temporal characteristics (e.g., age, maturational level, etc.) and their potential use as a specifier of early specialization. The results of this study support consideration of maturation status in future efforts to educate athletes, parents, and coaches regarding sport specialization. They may also provide guidance on the inclusion of integrative neuromuscular training programs for young females who chose to specialize early.

## Ethics Statement

Prior to data collection in both sessions, the study protocol was approved by the Cincinnati Children's Hospital Medical Center Institutional Review Board, and informed written consent, along with child assent, in accordance with the Declaration of Helsinki, was obtained from participants and their parents or legal guardians if under 18 years of age.

## Author Contributions

CD was responsible for the overall study design, analysis, preparation, and writing of the manuscript. AM was responsible for the preparation and writing of the manuscript. KB, ST, KF, GM, and TH were responsible for data collection and oversight, and preparation and writing of the manuscript. GM was also responsible for overall concept and study design. NJ, AS, and DB were responsible for preparation and writing of the manuscript.

### Conflict of Interest Statement

The authors declare that the research was conducted in the absence of any commercial or financial relationships that could be construed as a potential conflict of interest.

## References

[1] Jayanthi N Pinkham C Dugas L Patrick B Labella C. Sports specialization in young athletes: evidence-based recommendations. Sports Health. (2013) 5:251–7. 10.1177/1941738112464626 24427397 PMC3658407

[2] Bell DR Post EG Trigsted SM Hetzel S McGuine TA Brooks MA. Prevalence of sport specialization in high school athletics: a 1-year observational study. Am J Sports Med. (2016) 44:1469–74. 10.1177/0363546516629943 26920433

[3] Strum R Childhood obesity—what we can learn from existing data on societal trends, part 1. Prevent Chron Dis. (2005) 2:A12 Available online at: https://www.cdc.gov/pcd/issues/2005/jan/04_0038.htm PMC1323315 15670465

[4] Gould D. The professionalization of youth sports: it's time to act! Clin J Sport Med. (2009) 19:81–2. 10.1097/JSM.0b013e31819edaff 19451759

[5] Carlson R The socialization of elite tennis players in Sweden: an analysis of the players' backgrounds and development. Sociol Sport J. (1988) 5:241–56. 10.1123/ssj.5.3.241

[6] Güllich A Emrich E Evaluation of the support of young athletes in the elite sports system. Eur J Sport Soc. (2006) 3:85–108. 10.1080/16138171.2006.11687783

[7] Malina RM. Early sport specialization: roots, effectiveness, risks. Curr Sports Med Rep. (2010) 9:364–71. 10.1249/JSR.0b013e3181fe3166 21068571

[8] Baxter-Jones AD Maffulli N Group TS. Parental influence on sport participation in elite young athletes. J Sports Med Phys Fitness. (2003) 43:250–5. 12853909

[9] LaPrade RF Agel J Baker J Brenner JS Cordasco FA Côté J. AOSSM early sport specialization consensus statement. Orthop J Sports Med. (2016) 4:2325967116644241. 10.1177/2325967116644241 27169132 PMC4853833

[10] Brenner JS American Academy of Pediatrics Council on Sports Medicine and Fitness. Overuse injuries, overtraining, and burnout in child and adolescent athletes. Pediatrics. (2007) 119:1242–5. 10.1542/peds.2007-0887 17545398

[11] DiFiori JP Benjamin HJ Brenner JS Gregory A Jayanthi N Landry GL. Overuse injuries and burnout in youth sports: a position statement from the American Medical Society for Sports Medicine. Br J Sports Med. (2014) 48:287–8. 10.1136/bjsports-2013-093299 24463910

[12] Lopiano DA. Modern history of women in sports. twenty-five years of Title IX. Clin Sports Med. (2000) 19:163–73. 10.1016/S0278-5919(05)70196-4 10740752

[13] Post EG Trigsted SM Riekena JW Hetzel S McGuine TA Brooks MA. The association of sport specialization and training volume with injury history in youth athletes. Am J Sports Med. (2017) 45:1405–12. 10.1177/0363546517690848 28288281

[14] McGuine TA Post EG Hetzel SJ Brooks MA Trigsted S Bell DR. A prospective study on the effect of sport specialization on lower extremity injury rates in high school athletes. Am J Sports Med. (2017) 45:2706–12. 10.1177/0363546517710213 28735552

[15] Myer GD Jayanthi N Difiori JP Faigenbaum AD Kiefer AW Logerstedt D. Sport specialization, part i: does early sports specialization increase negative outcomes and reduce the opportunity for success in young athletes? Sports Health. (2015) 7:437–42. 10.1177/1941738115598747 26502420 PMC4547120

[16] Feeley BT Agel J LaPrade RF. When is it too early for single sport specialization? Am J Sports Med. (2016) 44:234–41. 10.1177/0363546515576899 25825379

[17] Jayanthi NA LaBella CR Fischer D Pasulka J Dugas LR. Sports-specialized intensive training and the risk of injury in young athletes a clinical case-control study. Am J Sports Med. (2015) 43:794–801. 10.1177/0363546514567298 25646361

[18] Hall R Barber Foss K Hewett TE Myer GD. Sport specialization's association with an increased risk of developing anterior knee pain in adolescent female athletes. J Sport Rehabil. (2015) 24:31–5. 10.1123/jsr.2013-0101 24622506 PMC4247342

[19] Hamill J Palmer C Van Emmerik RE. Coordinative variability and overuse injury. Sports Med Arthrosc Rehabil Ther Technol. (2012) 4:45. 10.1186/1758-2555-4-45 23186012 PMC3536567

[20] Olsen SJ 2nd Fleisig GS Dun S Loftice J Andrews JR. Risk factors for shoulder and elbow injuries in adolescent baseball pitchers. Am J Sports Med. (2006) 34:905–12. 10.1177/0363546505284188 16452269

[21] Abrams GD Renstrom PA Safran MR. Epidemiology of musculoskeletal injury in the tennis player. Br J Sports Med. (2012) 46:492–8. 10.1136/bjsports-2012-091164 22554841

[22] Fransen J Pion J Vandendriessche J Vandorpe B Vaeyens R Lenoir M. Differences in physical fitness and gross motor coordination in boys aged 6-12 years specializing in one versus sampling more than one sport. J Sports Sci. (2012) 30:379–86. 10.1080/02640414.2011.642808 22214429

[23] Myer GD Jayanthi N DiFiori JP Faigenbaum AD Kiefer AW Logerstedt D. Sports specialization, part II: alternative solutions to early sport specialization in youth athletes. Sports Health. (2016) 8:65–73. 10.1177/1941738115614811 26517937 PMC4702158

[24] Myer GD Ford KR Palumbo JP Hewett TE. Neuromuscular training improves performance and lower-extremity biomechanics in female athletes. J Strength Condition Res. (2005) 19:51–60. 10.1519/00124278-200502000-00010 15705045

[25] Myer GD Ford KR McLean SG Hewett TE. The effects of plyometric versus dynamic stabilization and balance training on lower extremity biomechanics. Am J Sports Med. (2006) 34:445–55. 10.1177/0363546505281241 16282579

[26] Csintalan RP Inacio MC Funahashi TT. Incidence rate of anterior cruciate ligament reconstructions. Perm J. (2008) 12:17–21. 10.7812/TPP/07-140 21331205 PMC3037119

[27] Agel J Arendt EA Bershadsky B. Anterior cruciate ligament injury in national collegiate athletic association basketball and soccer: a 13-year review. Am J Sports Med. (2005) 33:524–30. 10.1177/0363546504269937 15722283

[28] Hewett TE Myer GD Ford KR Decrease in neuromuscular control about the knee with maturation in female athletes. J Bone Joint Surg Am Vol. (2004) 86a:1601–8. 10.2106/00004623-200408000-00001 15292405

[29] Hewett TE Myer GD Ford KR Heidt RS Jr. Colosimo AJ McLean SG. Biomechanical measures of neuromuscular control and valgus loading of the knee predict anterior cruciate ligament injury risk in female athletes: a prospective study. Am J Sports Med. (2005) 33:492–501. 10.1177/0363546504269591 15722287

[30] Ford KR Myer GD Schmitt LC Uhl TL Hewett TE. Preferential quadriceps activation in female athletes with incremental increases in landing intensity. J Appl Biomech. (2011) 27:215–22. 10.1123/jab.27.3.215 21844610 PMC4221795

[31] Quatman CE Ford KR Myer GD Hewett TE. Maturation leads to gender differences in landing force and vertical jump performance - A longitudinal study. Am J Sports Med. (2006) 34:806–13. 10.1177/0363546505281916 16382009

[32] Pappas E Shiyko MP Ford KR Myer GD Hewett TE. Biomechanical deficit profiles associated with ACL injury risk in female athletes. Med Sci Sports Exerc. (2016) 48:107–13. 10.1249/MSS.0000000000000750 26258858 PMC4681676

[33] Myer GD Ford KR Divine JG Wall EJ Kahanov L Hewett TE. Longitudinal assessment of noncontact anterior cruciate ligament injury risk factors during maturation in a female athlete: a case report. J Athletic Train. (2009) 44:101–9. 10.4085/1062-6050-44.1.101 19180226 PMC2629034

[34] Ford KR Myer GD Hewett TE. Longitudinal effects of maturation on lower extremity joint stiffness in adolescent athletes. Am J Sports Med. (2010) 38:1829–37. 10.1177/0363546510367425 20522830 PMC3968426

[35] Ford KR Shapiro R Myer GD Van Den Bogert AJ Hewett TE. Longitudinal sex differences during landing in knee abduction in young athletes. Med Sci Sports Exerc. (2010) 42:1923–31. 10.1249/MSS.0b013e3181dc99b1 20305577 PMC2924455

[36] Myer GD Ford KR Barber Foss KD Goodman A Ceasar A Rauh MJ. The incidence and potential pathomechanics of patellofemoral pain in female athletes. Clin Biomech. (Bristol, Avon). (2010) 25:700–7. 10.1016/j.clinbiomech.2010.04.001 20466469 PMC2900391

[37] Hewett TE Ford KR Xu YYY Khoury J Myer GD. Utilization of ACL injury biomechanical and neuromuscular risk profile analysis to determine the effectiveness of neuromuscular training. Am J Sport Med. (2016) 44:3146–51. 10.1177/0363546516656373 27474385 PMC5513480

[38] Davies PL Rose JD Motor skills of typically developing adolescents: awkwardness or improvement? Phys Occup Ther Pediatr. (2000) 20:19–42. 10.1080/J006v20n01_03 11293913

[39] Davies PL Rose JD Assessment of cognitive development in adolescents by means of neuropsychological tasks. Dev Neuropsychol. (1999) 15:227–48. 10.1080/87565649909540747

[40] Galloway RT Xu Y Hewett TE Barber Foss K Kiefer AW DiCesare CA. Age-dependent patellofemoral pain: hip and knee risk landing profiles in prepubescent and postpubescent female athletes. Am J Sports Med. (2018) 46:2761–71. 10.1177/0363546518788343 30091937 PMC9709661

[41] Ford KR Myer GD Hewett TE. Reliability of landing 3D motion analysis: implications for longitudinal analyses. Med Sci Sports Exerc. (2007) 39:2021–8. 10.1249/mss.0b013e318149332d 17986911

[42] Paterno MV Schmitt LC Ford KR Rauh MJ Myer GD Huang B. Biomechanical measures during landing and postural stability predict second anterior cruciate ligament injury after anterior cruciate ligament reconstruction and return to sport. Am J Sports Med. (2010) 38:1968–78. 10.1177/0363546510376053 20702858 PMC4920967

[43] Sigward SM Pollard CD Powers CM. The influence of sex and maturation on landing biomechanics: implications for anterior cruciate ligament injury. Scand J Med Sci Sports. (2012) 22:502–9. 10.1111/j.1600-0838.2010.01254.x 21210853 PMC3117023

[44] Ford KR Myer GD Brent JL Hewett TE. Hip and knee extensor moments predict vertical jump height in adolescent girls. J Strength Condition Res Nat Strength Condition Assoc. (2009) 23:1327–31. 10.1519/JSC.0b013e31819bbea4 19528842 PMC4010199

[45] Côté J The influence of the family in the development of talent in sport. Sport Psychol. (1999) 13:395–417. 10.1123/tsp.13.4.395

[46] Wiersma LD Risks and benefits of youth sport specialization: perspectives and recommendations. Pediatr Exerc Sci. (2000) 12:13–22. 10.1123/pes.12.1.13

[47] Baker J Cobley S Fraser-Thomas J What do we know about early sport specialization? Not much! High Abil Stud. (2009) 20:77–89. 10.1080/13598130902860507

[48] Côté J Lidor R Hackfort D To sample or to specialize? Seven postulates about youth sport activities that lead to continued participation and elite performance. Int J Sport Exerc Psychol. (2009) 9:7–17. 10.1080/1612197X.2009.9671889

